# Regeneration of collagen fibrils at the papillary dermis by reconstructing basement membrane at the dermal–epidermal junction

**DOI:** 10.1038/s41598-022-04856-1

**Published:** 2022-01-17

**Authors:** Shunsuke Iriyama, Yuki Ogura, Saori Nishikawa, Junichi Hosoi, Satoshi Amano

**Affiliations:** Shiseido Global Innovation Center, 1-2-11, Takashima, Nishi-ku, Yokohama, 220-0011 Japan

**Keywords:** Biochemistry, Cell biology, Developmental biology

## Abstract

The epidermal basement membrane deteriorates with aging. We previously reported that basement membrane reconstruction not only serves to maintain epidermal stem/progenitor cells in the epidermis, but also increases collagen fibrils in the papillary dermis. Here, we investigated the mechanism of the latter action. Collagen fibrils in the papillary dermis were increased in organotypic human skin culture treated with matrix metalloproteinase and heparinase inhibitors. The expression levels of *COL5A1* and *COL1A1* genes (encoding collagen type V α 1 chain and collagen type I α 1 chain, respectively) were increased in fibroblasts cultured with conditioned medium from a skin equivalent model cultured with the inhibitors and in keratinocytes cultured on laminin-511 E8 fragment-coated plates. We then examined cytokine expression, and found that the inhibitors increased the expression of PDGF-BB (platelet-derived growth factor consisting of two B subunits) in epidermis. Expression of *COL5A1* and *COL1A1* genes was increased in cultured fibroblasts stimulated with PDGF-BB. Further, the bifunctional inhibitor hydroxyethyl imidazolidinone (HEI) increased skin elasticity and the thickness of the papillary dermis in the skin equivalent. Taken together, our data suggests that reconstructing the basement membrane promotes secretion of PDGF-BB by epidermal keratinocytes, leading to increased collagen expression at the papillary dermis.

## Introduction

The papillary dermis, the uppermost layer of the dermis, is composed of loosely arranged fine collagen fibrils that intertwine with the anchoring fibrils at the basement membrane, thereby promoting dermal/epidermal adhesion^1^. Raman imaging has revealed changes in collagen fibrils at the papillary dermis with aging^2^, and histological studies similarly showed a reduction in the thickness of collagen bundles with age^3^. Type I/III collagen was also altered and decreased at the papillary dermis in photo-aged skin as compared with sun-protected skin of elderly people^4^. However, the relationship between basement membrane integrity and changes of the papillary dermal matrix remains unclear.

The epidermal basement membrane (BM) at the dermal–epidermal junction is a ubiquitous sheet-like structure that binds the dermis and the epidermis of the skin together. It is mainly composed of type IV and type VII collagens, laminin-511, laminin-332, nidogen, and perlecan^5^. The epidermal BM is damaged by several degradative enzymes, which are activated by UV irradiation and in sun-exposed skin^6,7^. The lamina densa at the dermal–epidermal junction is impaired and the anchoring fibrils that intertwine with the thin collagen fibrils at the papillary dermis are reduced in photoaged skin^8^. The corresponding gene expression levels in fibroblasts are similarly reduced^9^.

All of the above findings indicate that the interaction between the basement membrane and the dermis deteriorates with age. However, the precise role of the basement membrane in maintaining collagen fibrils in the papillary dermis remains unclear. Therefore, the aim of the present work was to characterize the age-dependent changes in the interaction between the basement membrane and the dermis, and to investigate the mechanism involved.

## Results

### Age-dependent changes of collagen fibrils in the papillary dermis

**Figure 1:**
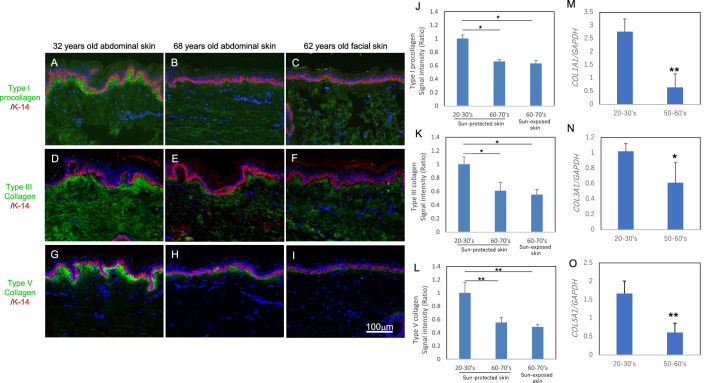
Age-dependent change of collagen fibrils in dermis. Immunostaining of type I procollagen (**A–C**), type III collagen (**D–F**) and type V collagen (**G–I**) in sun-protected young skin (**A, D, G**), sun-protected older skin (**B, E, H**) and sun-exposed older skin (**C, F, I**). Bars: 100 μm. Staining signal intensities of type I procollagen (**J**), type III collagen (**K**) and type V collagen (**L**) were analyzed using WINROOF 2015 image-analyzing software (Mitani, Fukui, Japan, https://www.mitani-visual.jp/products/image_analys_ismeasure-ment/winroof/). Data are the means ± SD of six images from six donors each in the 20–30’s and 60–70’s. * p < 0.05, ** p < 0.01. mRNA expression levels of *COL1A1* (**M**), *COL3A1* (**N**) and *COL5A1* (**O**) genes in cultured human fibroblasts from young and older donors were analyzed by qPCR. Data are the means ± SD of three independent experiments from six donors. *p < 0.05, **p < 0.01.

Collagen fibrils decrease with age in human skin, but the age-dependent changes of collagen fibrils in the papillary dermis have not been established. To investigate this, we performed immunostaining of type I procollagen and type III/V collagens in human skin from donors of different ages. Type I procollagen and type III/V collagens were stained beneath the basement membrane in the papillary dermis, and the staining intensities were lower in aged sun-protected skin and sun-exposed skin as compared with young sun-protected skin (Fig. 1A–L). Since the decrease of basement membrane component laminin-511 observed histologically was matched by a reduction of the gene expression with aging^10^, we next measured the expression levels of the *COL1A1*, *COL3A1* and *COL5A1* genes in cultured human fibroblasts from donors of different ages. Expression of these genes was reduced in fibroblasts from aged donors as compared with young donors (Fig. 1M–O).

### Increase of collagen fibrils in papillary dermis of an organotypic human skin model in the presence of MMP and heparinase inhibitors

**Figure 2:**
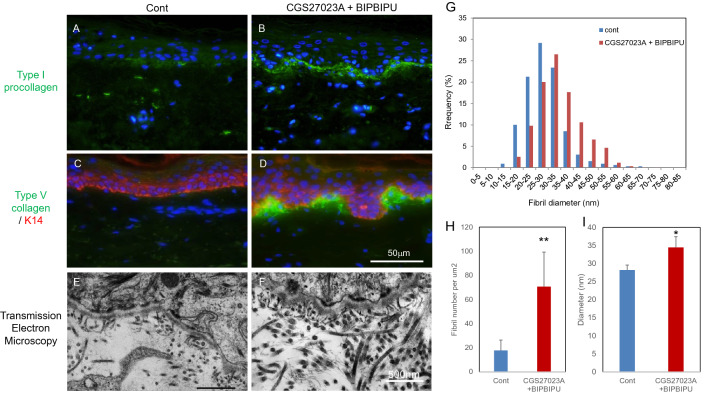
The effect of MMP inhibitor and heparinase inhibitor on the accumulation of collagen fibrils in the organotypic human skin model. Immunostaining of type I procollagen (**A, B**) and type V collagen (**C, D**), and transmission electron microscopy images (**E, F**) of organotypic human skin model in the presence of MMP inhibitor CGS27023A and heparinase inhibitor BIPBIPU (**B, D, F**) as compared with the control (**A, C, E**). The distribution (**G**), density (**H**) and diameter (**I**) of collagen fibrils were analyzed by WINROOF 2015. White bars in **D**: 50 μm. White bar in **F**: 500 nm. Data are expressed as mean ± SD of three independent experiments using samples from six donors. * p < 0.05, ** p < 0.01.

Histological and quantitative studies established that fibrillar collagens in the papillary dermis decrease with age and daily sun exposure (Fig. 1). We previously reported that the basement membrane was damaged as a result of activation of MMPs and heparinase in the epidermis after UV irradiation^6,7^, and treatment of organotypic human skin and skin equivalent models with both MMP and heparinase inhibitors promoted the formation of basement membrane at the dermal–epidermal junction and also resulted in the appearance of thicker collagen fibrils in the papillary dermis^11^. Therefore, we next exposed an organotypic human skin culture to these inhibitors and performed immunostaining for type I procollagen and type V collagen. Fluorescence microscopy revealed that the staining intensities of type I procollagen and type V collagen were increased in the papillary dermis after inhibitor treatment, as compared with the control (Fig. 2A–D). Thick collagen fibrils were observed beneath the basement membrane in the cultures treated with the inhibitors (Fig. 2E, F). We next evaluated the density and thickness of collagen fibrils in the papillary dermis using analytical software WINROOF2015. The density and thickness of collagen fibrils were increased significantly in the presence of both inhibitors as compared with the control (Fig. 2G–I).

### Increase of collagen fibrils in papillary dermis of the skin equivalent model in the presence of MMP and heparinase inhibitors

**Figure 3:**
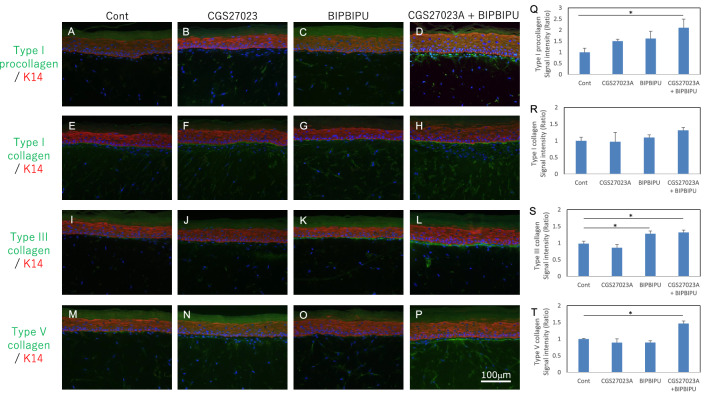
Effect of MMP inhibitor and heparinase inhibitor on collagen expression in the skin equivalent model. Immunostaining of type I procollagen (**A–D**), type I collagen (**E–H**), type III collagen (**I–L**) and type V collagen (**M–P**) in the skin equivalent model in presence of MMP inhibitor CGS27023A (**B, F, J, N**), heparinase inhibitor BIPBIPU (**C, G, K, O**), or both MMP inhibitor CGS27023A and heparinase inhibitor BIPBIPU (**D, H, L, P**) as compared with control (**A, E, I, M**). Staining signal intensities of type I procollagen (**Q**), type I collagen (**R**), type III collagen (**S**) and type V collagen (**T**) were analyzed using WINROOF 2015 image-analyzing software (Mitani, Fukui, Japan, https://www.mitani-visual.jp/products/image_analys_ismeasure-ment/winroof/). Data are the means ± SD of three independent experiments. *p < 0.05, **p < 0.01. White bars: 100 μm.

**Figure 4:**
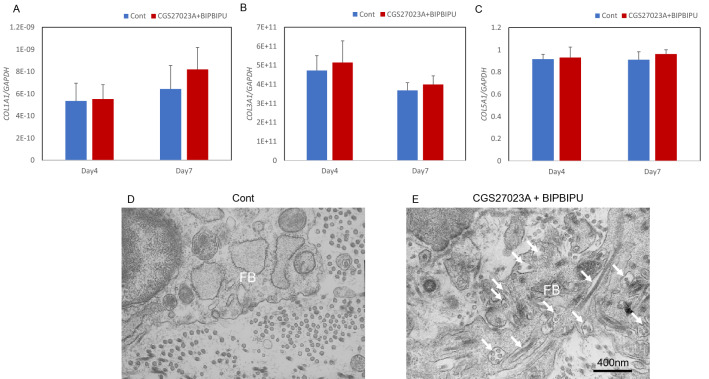
Effect of MMP inhibitor and heparinase inhibitor on the accumulation of collagen fibrils in the skin equivalent model. mRNA expression levels of the *COL1A1* (**A**), *COL3A1* (**B**) and *COL5A1* (**C**) genes were analyzed by qPCR. Data are expressed as mean ± SD of three independent experiments. The ultrastructure of collagen fibrils at the papillary dermis was imaged by transmission electron microscopy in the skin equivalent model in presence of MMP inhibitor CGS27023A and heparinase inhibitor BIPBIPU (**E**) as compared with control (**D**). Representative TEM images from three independent experiments are shown. FB: Fibroblasts. White arrow: collagen fibrils in fibroblasts. Black bar: 400 nm.

To further investigate the effects of the two inhibitors on the induction of collagen fibrils in the papillary dermis, we next cultured a skin equivalent model with each inhibitor or both, and performed immunostaining for type I procollagen and type I/III/V collagens. Type I and type V collagens were detected beneath the basement membrane at the papillary dermis, and the staining intensities of type V collagen were increased following treatment with both MMP and heparinase inhibitors as compared with the control (Fig. 3E–H, M–P, R, T). Type III collagen was also stained beneath the basement membrane at the papillary dermis and its intensity was increased following treatment with the heparinase inhibitor (Fig. 3I–L, S). Type I procollagen was stained in the cytoplasm of papillary fibroblasts beneath the basement membrane following treatment with both the MMP and heparinase inhibitors (Fig. 3A–D, Q). Next, we measured the expression levels of *COL1A1*, *COL3A1* and *COL5A1* genes in the dermis of the skin equivalent model. *COL1A1*, *COL3A1* and *COL5A1* gene expression levels were unchanged in the whole dermis following the inhibitor treatments (Fig. 4A–C). Since collagen deposition was increased locally beneath the basement membrane by the inhibitors, we examined this region by means of transmission electron microscopy and found that fibroblasts beneath the basement membrane actively secreted procollagen in the presence of the two inhibitors (Fig. 4D, E).

### Induction of collagen expression in the dermis via secretion of PDGF-BB in the epidermis following treatment with MMP and heparinase inhibitors

**Figure 5:**
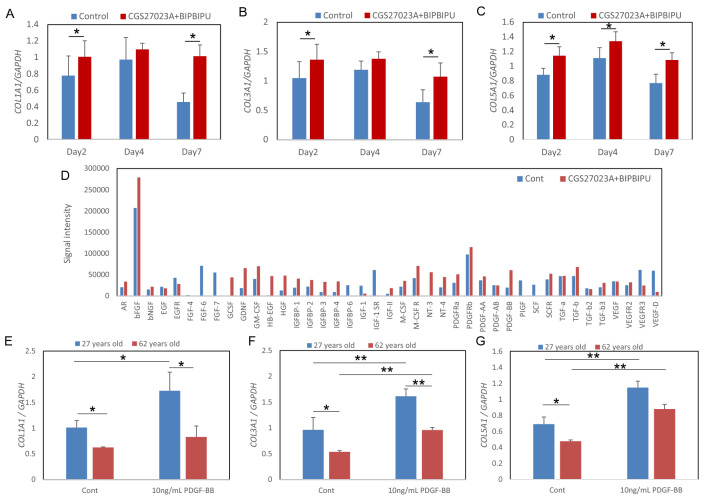
Promotion of collagen expression in fibroblasts by conditioned medium from the skin equivalent model. mRNA expression levels of the *COL1A1* (**A**), *COL3A1* (**B**) and *COL5A1* (**C**) genes in cultured fibroblasts cultured with conditioned media from the skin equivalent model were analyzed by qPCR. Data are expressed as mean ± SD of three independent experiments. * p < 0.05. Protein membrane array analysis was performed by using the cell lysate from epidermis of the skin equivalent model cultured with MMP inhibitor CGS27023A and heparinase inhibitor BIPBIPU (**D**). mRNA expression levels of the *COL1A1* (**E**), *COL3A1* (**F**) and *COL5A1* (**G**) genes in cytokine-stimulated fibroblasts were analyzed by qPCR. Data are expressed as mean ± SD of three independent experiments. *p < 0.05, **p < 0.01.

The above findings indicated that collagen may not only be protected, but also induced locally at the papillary dermis following treatment with the inhibitors (Figs. 2, 3, 4). We hypothesized that an unknown factor secreted from the epidermis—whose keratinocytes are attached to the basement membrane—acted on fibroblasts at the papillary dermis to promote collagen expression in the presence of both inhibitors. To test this idea, we next examined the effect of conditioned medium from the skin equivalent model cultured with or without both inhibitors on the expression of *COL1A1*, *COL3A1* and *COL5A1* genes in cultured fibroblasts. *COL1A1*, *COL3A1* and *COL5A1* expression levels were increased by the conditioned medium (Fig. 5A–C). Membrane array analysis to identify cytokines induced in the epidermis of the skin equivalent model by the inhibitors revealed that PDGF-BB, GDNF and GM-CSF were upregulated (Fig. 5D). Among these candidate cytokines, PDGF-BB significantly up-regulated *COL5A1* expression level in cultured fibroblasts (Supplementary Fig. S1). The expression levels of *COL1A1*, *COL3A1* and *COL5A1* were reduced in fibroblasts from a 67-year-old donor as compared with a 27-year-old donor, and were increased in presence of PDGF-BB in both cases (Fig. 5E–G). In addition, the staining intensities of type III and V collagens were increased in the dermis after treatment of PDGF-BB, as compared with the control, in the skin equivalent model (Supplementary Fig. S2).

### PDGFB expression in keratinocytes was reduced in an age-dependent manner and was affected by the basement membrane condition

**Figure 6:**
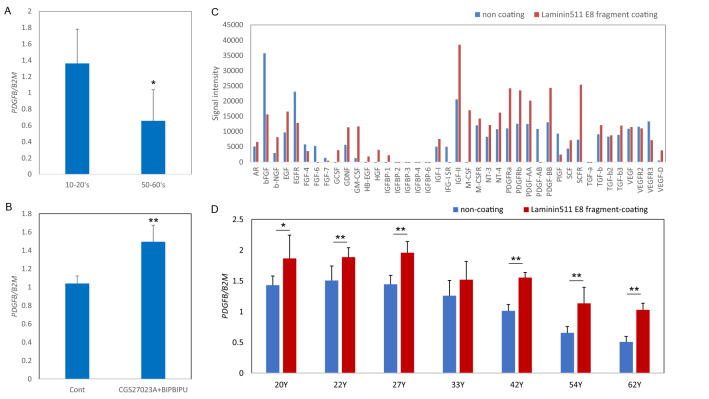
Age-dependent reduction of PDGFB expression and effect of laminin-511. mRNA expression levels of *PDGFB* in cultured keratinocytes from donors of different ages were analyzed by qPCR (**A**). Data are expressed as mean ± SD of three independent experiments from 6 donors each in the 10–20’s and 50–60’s. * p < 0.05. mRNA expression levels of *PDGFB* in epidermis of the skin equivalent model treated with or without MMP inhibitor CGS27023A and heparinase inhibitor BIPBIPU were analyzed by qPCR (**B**). Data are expressed as mean ± SD of three independent experiments. ** p < 0.01. Protein membrane array analysis was performed by using cell lysate from keratinocytes cultured on laminin-511 E8 fragment-coated or non-coated plates (**C**). mRNA expression levels of *PDGFB* in cultured keratinocytes from donors of different ages cultured on laminin-511 E8 fragment-coated or non-coated plates (**D**) were analyzed by qPCR. Data are expressed as mean ± SD of three independent experiments. *p < 0.05, **p < 0.01.

To investigate the age-dependent change of *PDGFB* expression in epidermis, we measured the expression levels of the *PDGFB* gene in cultured keratinocytes from donors of different ages. *PDGFB* expression in keratinocytes was lower in older donors (Fig. 6A). In addition, the *PDGFB* expression level in epidermis of the skin equivalent model was increased in the presence of both MMP inhibitor and heparinase inhibitor (Fig. 6B). We previously reported that epidermal stem cell levels were maintained by blocking the degradation of laminin-511 mediated by MMP-9 and heparinase^10^. Thus, we next investigated the effect of laminin-511 on cytokine secretion. Protein levels of not only PDGF-BB but also GDNF and GM-CSF were increased in keratinocytes cultured on laminin-511 E8 fragment-coated plates, as compared with non-coated plates (Fig. 6C), in accordance with the protein array data for epidermis of the skin equivalent model (Fig. 5D). The mRNA expression level of *PDGFB* in keratinocytes cultured on laminin-511 E8 fragment-coated plates was increased as compared with those on non-coated plates for donors of all ages (Fig. 6D). In addition, the expression levels of *COL1A1*, *COL3A1* and *COL5A1* genes were increased by stimulation with conditioned medium from keratinocytes cultured on a laminin 511 E8 fragment coating (Supplementary Fig. S3A–C). These data indicated that preventing the degradation of laminin-511 is critical to promote PDGF-BB expression in keratinocytes.

### PDGFR-beta-positive fibroblasts were located beneath the basement membrane in papillary dermis of young skin and aged skin

PDGF-BB binds to the two structurally related PDGF receptors (PDGFR-α and PDGFR-β). PDGFR-α-positive cells are located in reticular and papillary dermis^11,12^ and PDGFR-β-positive cells are located in papillary dermis^13^ in mice.

**Figure 7:**
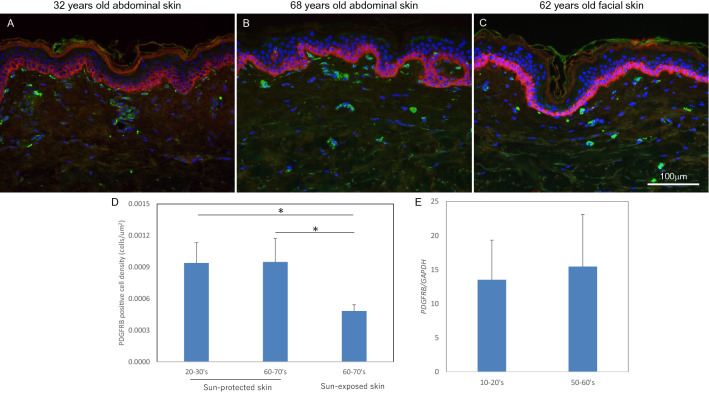
Localization of PDGFR-beta positive cells in human skin. Immunostaining of PDGFRβ in young sun-protected skin (**A**), older sun-protected skin (**B**) and older sun-exposed skin (**C**). PDGFRβ-positive cell density was analyzed by using WINROOF 2015 (**D**). Bars: 100 μm. Data are the means ± SD of six images from each of six donors. mRNA expression level of *PDGFRB* in cultured fibroblasts was analyzed by qPCR (**E**). Data are expressed as mean ± SD of six independent experiments on samples from six donors.

To investigate the location of PDGFR-β-positive fibroblasts in human skin, we performed immunostaining for PDGFR-β using human skin from donors of different ages. PDGFR-β-positive cells were observed beneath the basement membrane and at vessels in the papillary dermis in sun-protected young skin, sun-protected older skin and sun-exposed older skin (Fig. 7A–C). The density of PDGFR-β-positive cells was reduced in sun-exposed older skin as compared with sun-protected young or older skin (Fig. 7D). Further, the expression level of the *PDGFRB* gene in cultured fibroblasts showed no change with aging (Fig. 7E).

### Effect of bifunctional inhibitor of MMPs and heparinase on collagen expression at the papillary dermis in skin equivalent model and organotypic human skin model

The structure of the BM was protected not only by inhibitors of MMPs and heparinase, but also by hydroxyethyl imidazolidinone (HEI), which inhibits the enzymatic activities of both MMP-9 and heparinase^14^. We hypothesized that collagen expression is increased at the papillary dermis because the bifunctional inhibitor HEI blocks degradation of the basement membrane at the dermal–epidermal junction, leading to increased PDGF-BB expression in the epidermis. To test this idea, we next examined the expression levels of PDGFR-β, type I procollagen, and type V collagen in the skin equivalent model and the organotypic human skin model treated with HEI. PDGFR-β-positive cells were observed beneath the basement membrane in papillary dermis treated or not treated with inhibitors (Supplementary Fig. S4A–C, S5A-C). Type I procollagen and type V collagen were stained beneath the basement membrane at the papillary dermis, and the staining intensities were increased by treatment with HEI or both MMP inhibitor and heparinase inhibitor (Supplementary Figs. S4D–I, S5D–I).

### Effect of bifunctional inhibitor of MMPs and heparinase on accumulation of extracellular matrix at the papillary dermis of facial skin

**Figure 8:**
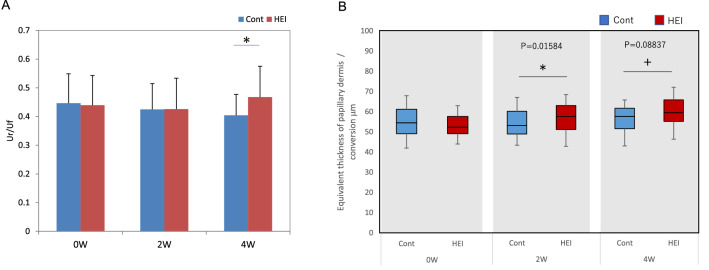
Improving effect of HEI blended lotion on elasticity of facial skin. The skin elasticity was measured with a cutometer, before and after treatment with HEI or with placebo control (**A**). The thickness of the papillary dermis was measured by scanning acoustic microscopy (**B**). *; P < 0.05.

We further investigated the effect of the bifunctional inhibitor HEI on collagen accumulation at the papillary dermis by applying lotion containing 1.5% HEI twice a day for 2 and 4 weeks to facial skin of human volunteers. HEI significantly increased the water content in the stratum corneum of facial skin at 2 and 4 weeks and reduced TEWL at 4 weeks, as compared with controls (Supplementary Fig. S6A, B), in agreement with previous findings^14^. The elasticity of facial skin was significantly increased at 4 weeks as compared with the control (Fig. 8A). Furthermore, the thickness of the papillary dermis in facial skin was significantly increased at 2 weeks and was also increased, though not significantly, at 4 weeks as determined by scanning acoustic microscopy (Fig. 8B).

## Discussion

In this study, we confirmed an aging-related decrease of collagens in human sun-protected skin, as well as sun-exposed skin (Fig. 1A–I). Furthermore, the gene expression levels of *COL1A1*, *COL3A1* and *COL5A1* were reduced in fibroblasts from aged donors as compared with young donors (Fig. 1M–O). On the other hand, collagen fibrils were increased at the papillary dermis in the presence of MMP inhibitor, CGS27023A and heparinase inhibitor, BIPBIPU in both the organotypic human skin model (Fig. 2) and the skin equivalent model (Figs. 3, 4). CGS27023A and BIPBIPU did not affect *COL1A1*, *COL3A1* and *COL5A1* gene expression (Fig. 3G–I). CGS27023A, which inhibits MMP-2, MMP-8 and MMP-9^15^, augmented deposition of type IV collagens at the dermal–epidermal junction, resulting in the formation of continuous epidermal BM^16^. Therefore, since collagens are degraded by MMPs, the increase of collagens appears to be due to decreased degradation in the presence of CGS27023A. However, type I/V collagens were increased in the skin equivalent model in the presence of CGS27023A (Fig. 3), whereas type III collagen was increased by BIPBIPU, but not CGS27023A. In addition, type I pro-collagen was stained in the cytoplasm of papillary fibroblasts in the presence of both CGS27023A and BIPBIPU (Fig. 3). The antibodies for type I collagen and type I pro-collagen responded only to newly synthesized collagen from human fibroblasts, and were not reactive with the guinea pig collagen used to prepare the skin equivalent model. Furthermore procollagen secreted by fibroblasts was detected beneath the basement membrane at the papillary dermis in the presence of both CGS27023A and BIPBIPU (Fig. 4D, E). These data suggest that expression and secretion of procollagen were locally promoted beneath the basement membrane at the papillary dermis in the presence of both CGS27023A and BIPBIPU.

Type I procollagen and type V collagen fibrils were accumulated beneath the basement membrane, but were reduced with aging (Fig. 1). Our present findings show that PDGFR-β-positive fibroblasts were decreased in sun-exposed older skin as compared with sun-protected young or older skin (Fig. 7A–D). On the other hand, PDGFB expression was reduced with aging in cultured human keratinocytes (Fig. 6A). PDGF-BB is known to be synthesized by keratinocytes^17^ and is involved in wound repair by promoting type I collagen expression in the dermis^18^. Furthermore, platelet-rich plasma (PRP) is used clinically for many purposes, as it contains abundant growth factors, including PDGF-BB^19,20^. PDGF-BB interacts with both α- and β-receptor subtypes to promote self-renewal of mesenchymal stem cells^21^ and collagen expression^22^ during wound closure^23^. PDGFRβ is expressed in fibroblasts throughout the dermis in juveniles and at the papillary dermis in adults, and the upper lineages of papillary fibroblasts play an important role as mesenchymal progenitors during wound healing^11,24^. In addition, topical application of recombinant human PDGF-BB promoted type III/V collagen expression in whole dermis of the human skin equivalent model (Supplementary Fig. S2). On the other hand, treatment with MMPs inhibitor and heparinase inhibitor enhanced type I/III/V collagen levels in papillary dermis of the skin equivalent model. These data suggest that PDGF-BB produced in the epidermis is transferred into the dermis, and cross-talk between PDGFR-β-positive fibroblasts and basal keratinocytes orchestrates collagen expression in the papillary dermis.

PDGF-BB expression was increased in epidermis of the skin equivalent model by treatment with both inhibitors (Fig. 6B), and was also increased in cultured human keratinocytes grown on plates coated with laminin-511 E8 fragment (Fig. 6C, D). We previously reported that laminin-511 was reduced with aging and sun exposure^10^. Since MMPs and heparinase were activated in epidermis of the skin equivalent model, as in UVB-exposed human skin^25,26^, treatment with MMPs inhibitor and heparinase inhibitor would suppress degradation of the basement membrane, and thereby promote deposition of laminin-511 at the dermal–epidermal junction^10^. These data suggest that PDGF-BB was induced in epidermis as a consequence of MMPs inhibitor- and heparinase inhibitor-mediated suppression of the decrease of laminin-511. Further work to elucidate the mechanism through which laminin-511 promotes PDGF-BB expression in epidermis is in progress.

We previously developed HEI as a bi-functional inhibitor of both MMP-9 and heparinase. Topical application of HEI promoted reconstruction of the basememt membrane and deposition of laminin-511 by inhibiting the activities of MMP-9 and heparinase in an organotypic human skin model^14^, thereby maintaining MCSP-positive epidermal stem cells and promoting epidermal homeostasis and barrier recovery^10,14^. The elasticity of facial skin was significantly increased at 4 weeks by treatment with HEI (Fig. 8A). In addition, the thickness of papillary dermis was increased significantly at 2 weeks and was also increased at 4 weeks, though not significantly (Fig. 8B). These in vivo human data suggest that collagen at the papillary dermis is increased in the presence of HEI via induction of PDGF-BB in the epidermis, resulting in stimulation of PDGFR-β-positive fibroblasts.

## Materials and methods

### Subjects

Skin samples from subjects between the ages of 26 and 78 years were obtained from Biopredic International, Rennes, France. Studies were conducted in accordance with the principles of the Declaration of Helsinki, and all participants had given written, informed consent to provide samples for research. The experimental protocols were also approved by ethics committee at Shiseido. The characteristics of the donors are summarized in Supplementary Table S1. Skin samples were fixed in cold acetone (AMeX procedure) and embedded in paraffin for immunohistochemical observation using specific antibodies.

### Materials

Heparanase inhibitor 1-[4-(1H-benzoimidazol-2-yl)phenyl]-3-[4-(1H-benzo-imidazol-2-yl)phenyl]urea (BIPBIPU), MMP inhibitor N-hydroxy-2(R)-[[(4-methoxyphenyl)sulfonyl](3-picolyl)amino]-3-methylbutanamide hydrochloride (CGS27023A), and bi-functional inhibitor hydroxyethyl imidazolidinone (HEI) were synthesized at Shiseido Co. Ltd. (Yokohama, Japan) according to the reported methods^14,27,28^.

### Skin equivalent (SE) model

The SE model (EFT-400) and medium (EFT-400-ASY) were purchased from MatTek Corp. (Ashland, MA). The SE model was cultured in the presence or absence of 0.1 mg/mL HEI, BIPBIPU (10^–5^ M) and/or CGS27023A (10^–5^ M) as described^14^. At 4 days after the start of culture, samples were fixed in cold acetone (AMeX procedure) and embedded in paraffin for immunohistochemical analysis. At 4 days after the start of culture, samples were separated into epidermis and dermis, and RNA was extracted for quantitative PCR analysis^14^.

### Organotypic human skin model

Fresh human abdominal skin (from 4 females aged 22–32; the characteristics of the donors are summarized in Supplementary Table S2) (Biopredic International, Rennes, France) was cut into 1.5 × 1.5 cm pieces, which were cultured in Humedia-KG2:DMEM (1:1) medium with magnesium ascorbyl phosphate (0.2 mM) in the presence or absence of 0.1 mg/mL HEI, BIPBIPU (10^–5^ M) and CGS27023A (10^–5^ M) after UVB irradiation (50 mJ/cm^2^) as described^14^. At 5 days after the start of culture, samples were fixed in cold acetone (AMeX procedure) and embedded in paraffin for immunostaining^14^.

### Immunohistochemistry

Each section was incubated overnight at 4 °C with primary antibodies targeting Type I procollagen (ab64409, rat mAb, Abcam, Cambridge, UK, cross-reacts with human), Type I collagen (600–401-103, rabbit pAb, Rockland, Limerick, PA, cross-reacts with bovine and human), Type III collagen (ab6310, FH-7A, mouse mAb, Abcam, Cambridge, UK, cross-reacts with rat and human), Type V collagen (AM10159PU-N, V13F6, mouse mAb, Acris, San Diego, CA, cross-reacts with human), keratin-14 (20R-CP002, guinea pig pAb, Fitzgerald Industries International, Acton, MA) and PDGFRbeta (MAB1263, mouse mAb, R&D Systems, Minneapolis, MN). Primary antibodies were detected using Alexa488- or Alexa594-conjugated secondary antibody (Thermo Fisher Scientific). Sections were examined with an Olympus BX51 microscope (Olympus, Tokyo, Japan) and images were captured with a DP72 controller digital camera (Olympus). The staining intensity in 6 randomly selected microscopic fields was quantified by using WINROOF2015 image-analyzing software (Mitani, Fukui, Japan, https://www.mitani-visual.jp/products/image_analys_ismeasurement/winroof/), as described previously^10^.

### Transmission electron microscopy

After 4-day culture (skin equivalent model) or 5-day culture (organotypic abdominal skin model), samples were immersed in glutaraldehyde fixative at 4 °C overnight and then rinsed in 0.1 M phosphate buffer for 30 min at room temperature as described^10^. Samples were fixed in 1% OsO_4_ for 30 min at room temperature and then rinsed in triple-distilled water, embedded and subjected to routine processing. Finally, thin sections were stained with uranyl acetate and lead citrate and examined with an electron microscope (JEM-1400; JEOL Ltd., Tokyo, Japan)^10^. TEM images showing 30 to 170 collagen fibrils were acquired, and the fibril diameter was analyzed from 10 images in each group in three independent experiments by using WINROOF2015 image-analyzing sofware (Mitani, Fukui, Japan, https://www.mitani-visual.jp/products/image_analys_ismeasurement/winroof/).

### Cell culture

Human normal epidermal keratinocytes from donors of various ages (for details, see Supplementary Table S3) (Biopredic International, Rennes, France) were cultured in Humedia-KG2 (KURABO, Osaka, Japan). Human dermal fibroblasts from donors of various ages (for details, see Supplementary Table S4) (Biopredic International, Rennes, France) were cultured in DMEM containing 10% FBS (Gibco, Tokyo, Japan). Culture flasks were incubated at 37 °C in a humidified atmosphere with 5% CO_2_. 2.5 × 10^5^ keratinocytes or fibroblasts were seeded into 6-well plates coated or not coated with iMatrix-511, and incubated for 24 h. RNA was extracted from each sample for quantitative PCR analysis and protein was extracted using RIPA lysis buffer (Nacalai tesque, Kyoto, Japan) for protein array analysis.

### Quantitative real-time RT-PCR

Total RNAs from cultured keratinocytes, fibroblasts, and separated epidermis and dermis from the skin equivalent model were isolated using the Qiagen Rneasy mini kit (Qiagen) and cDNA was synthesised using SuperScript VILO cDNA Synthesis Kit (Thermo Fisher Scientific). Expression of *COL5A1*, *COL3A1*, *COL1A1*, *PDGFB*, *PDGFRB*, *B2M* and *GAPDH* genes was analyzed by means of quantitative PCR using Platinum SYBR Green qPCR superMix-UDG (Invitrogen Japan, Tokyo, Japan), as described^25^. Primer sequences used were as follows: *COL5A1* forward; 5’-GTGGCACAGAATTGCTCTCA-3’, *COL5A1* reverse; 5’-TCACCCTCAAACACCTCCTC-3’, PDGFRb forward; 5’-CCTCAT-CATGCTTTGGCAGAAGAA-3’, *PDGFRB* reverse; 5’-GCTCATGTCCATGTA-GCCACCGTC-3’, *COL3A1* forward; 5’-TCCGGGTGAGAAAGGTGA-3’, *COL3A1* reverse; 5’-GCAGGTCCAGAACCTCCAG-3’, *COL1A1* forward; 5’-C-TCGAGGTGGACACCACCCT-3’, *COL1A1* reverse; 5’-CAGCTGGATGGCCA-CATCGG-3’, *B2M* forward; 5’-GTGGGATCGAGACATGTAAGCA-3’, *B2M* reverse; 5’-CAATCCAAATGCGGCATCT-3’, *GAPDH* forward; 5’-GAAGGTG-AAGGTCGGAGTC-3’, *GAPDH* reverse; 5’-GAAGATGGTGATGGGATTTC-3’.

### Membrane protein array

After 4-day culture of the skin equivalent model in the presence of MMP inhibitor and heparinase inhibitor, the epidermis was separated from the dermis, and harvested using RIPA lysis buffer (Nacalai tesque, Kyoto, Japan). After 1-day culture on iMatrix-511 coated or non-coated plates, keratinocytes were harvested using RIPA lysis buffer. Protein concentration of the tissue lysates was normalized by using BCA protein assay (Nakarai, Tokyo). The levels of 41 cytokines in the tissue lysates were measured by using a membrane array (ab134002, Abcam, Cambridge, UK). Images were captured with a LAS-1000UVmini (FUJIFILM, Tokyo) and the signal intensity of each spot was calculated by analytical software, Multi Gauge Version 3.0 (FUJIFILM, Tokyo).

### Clinical study

Healthy Japanese female subjects (30–54 years old, n = 30) were enrolled in a single-blind study. HEI blended lotion was used to treat one side of the face, and placebo lotion was used on the other side. This study was carried out in March 2018 in Japan. It was conducted in accordance with the declaration of Helsinki protocols and was approved by the Ethics Committee of Shiseido (Approval number: B01471). The HEI-blended lotion or placebo lotion was applied twice a day for 4 weeks. Informed consent was obtained from all subjects before commencement of the study. Water-permeable barrier function of the cheek skin on each side was evaluated by measuring TEWL 3 times with a Vapometer at each time point. Hydration of the stratum corneum was also evaluated by measuring the skin conductance 3 times on each side with a Corneometer CM-825. Skin elasticity was evaluated by measuring Ur/Uf 3 times with a Cutometer. The thickness of the papillary dermis at the cheek was evaluated by scanning acoustic microscopy, as described^29^.

### Statistical analysis

Data are presented as mean values ± SD. Statistical significance was determined by analysis of variance (ANOVA) and P-values were calculated using Fisher’s protected least significant difference (Fisher’s PLSD) test.

## Supplementary Information

Supplementary Information 1.

Supplementary Information 2.

## Abbreviations

MMP(s): Matrix metalloproteinase(s)
DEJ: Dermal–epidermal junction
BM: Basement membrane
SE: Skin equivalent
OC: Organotypic culture
PDGF: Platelet-derived growth factor
PDGFR-β: Platelet-derived growth factor receptor-beta
