# Supplementary material for: Regeneration of collagen fibrils at the papillary dermis by reconstructing basement membrane at the dermal–epidermal junction
Source: Sci Rep. 2022 Jan 17;12:795. doi: 10.1038/s41598-022-04856-1 (PMC8764085; doi:10.1038/s41598-022-04856-1)
Supplement: Supplementary file 1 — Supplementary Information 1. [file 41598_2022_4856_MOESM1_ESM.pdf]

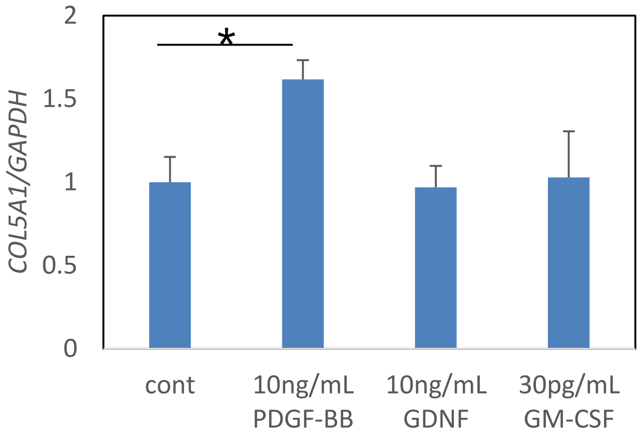
2
1.5
1
0.5
0
COL5A1/GAPDH
*
cont
10ng/mL PDGF-BB
10ng/mL GDNF
30pg/mL GM-CSF

Internal

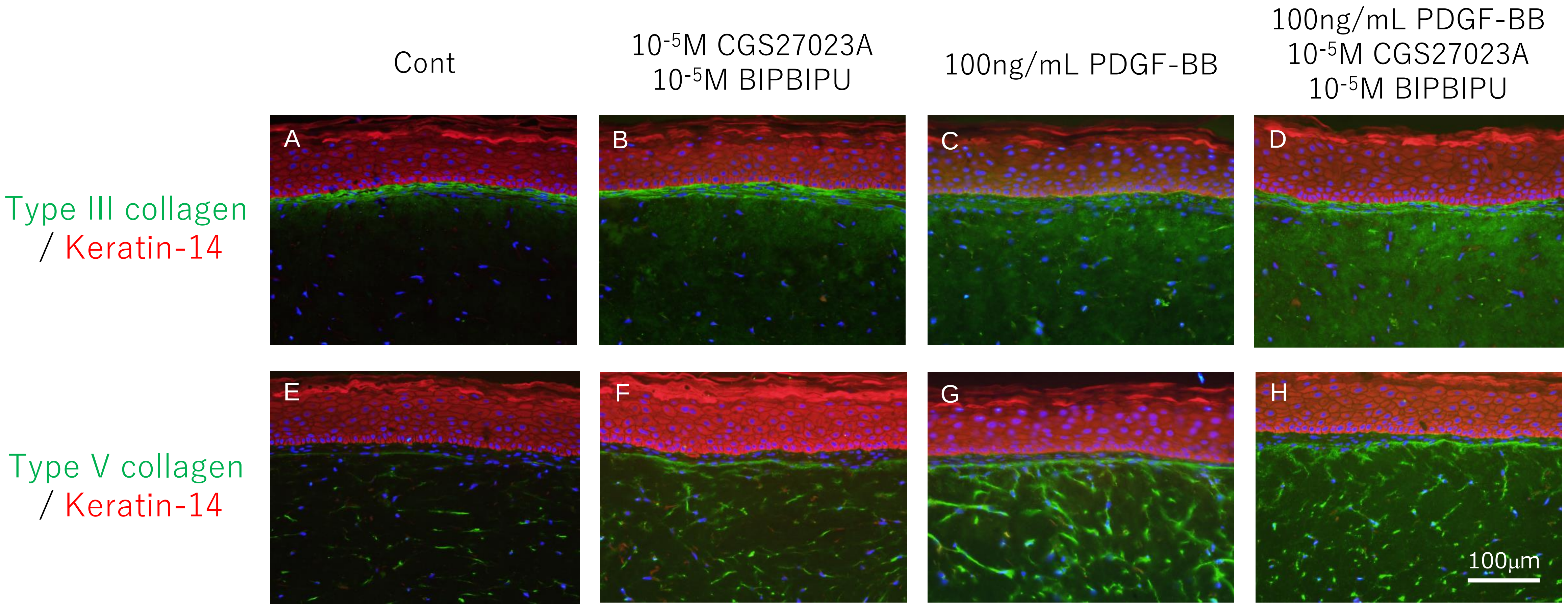

Cont
10-5M CGS27023A 10-5M BIPBIPU
100ng/mL PDGF-BB
100ng/mL PDGF-BB 10-5M CGS27023A 10-5M BIPBIPU
Type III collagen / Keratin-14
Type V collagen / Keratin-14
A
B
C
D
E
F
G
H
100μm

Internal

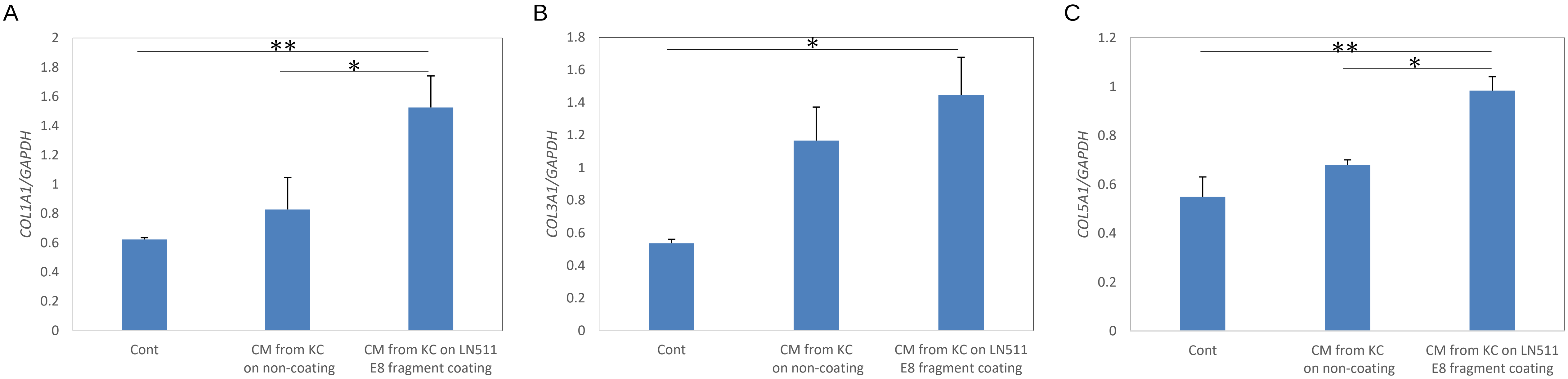

A
COL1A1/GAPDH
2
1.8
1.6
1.4
1.2
1
0.8
0.6
0.4
0.2
0
**
*
Cont
CM from KC
on non-coating
CM from KC on LN511
E8 fragment coating
B
COL3A1/GAPDH
1.8
1.6
1.4
1.2
1
0.8
0.6
0.4
0.2
0
*
Cont
CM from KC
on non-coating
CM from KC on LN511
E8 fragment coating
C
COL5A1/GAPDH
1.2
1
0.8
0.6
0.4
0.2
0
**
*
Cont
CM from KC
on non-coating
CM from KC on LN511
E8 fragment coating

Internal

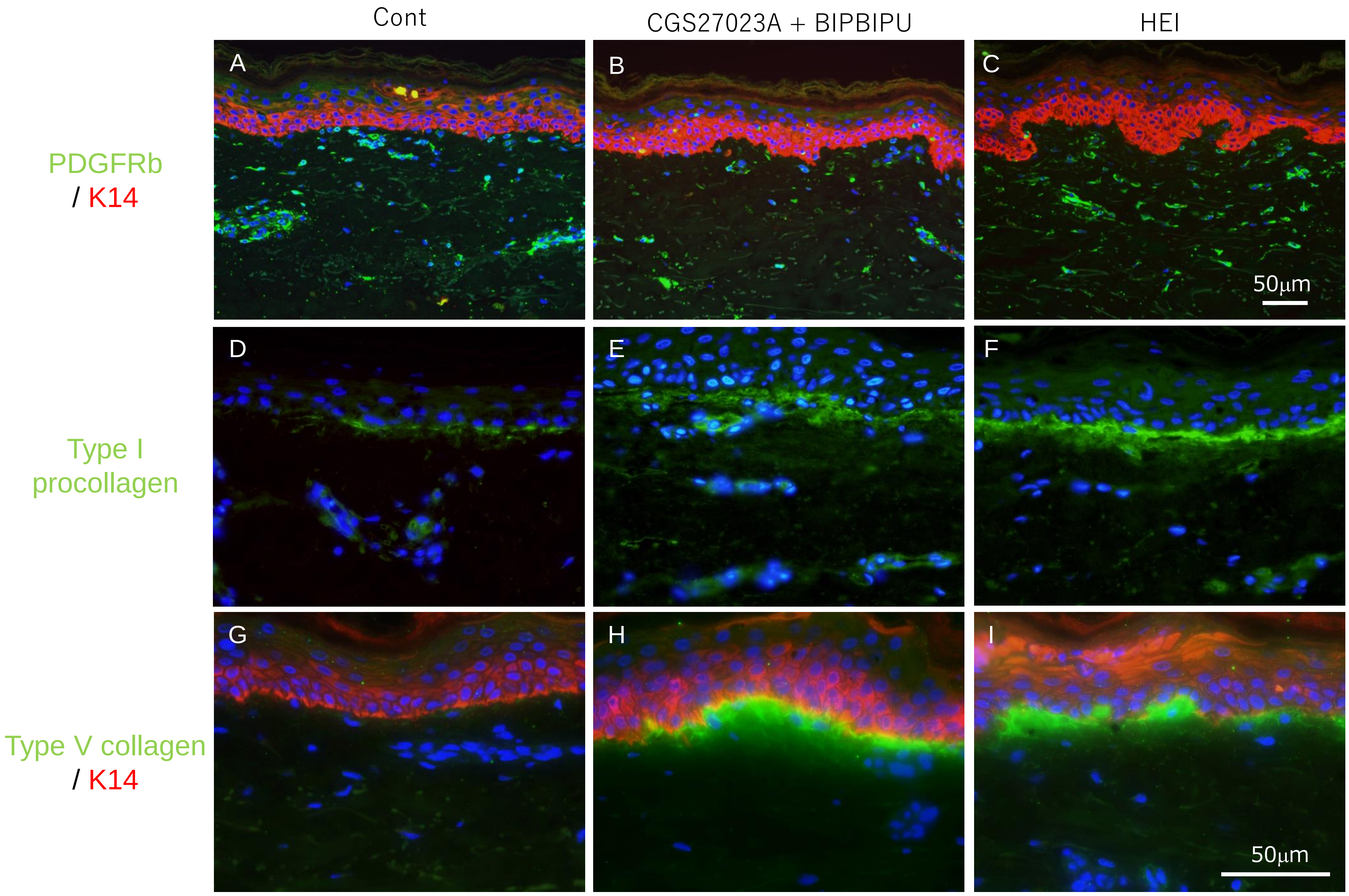

Cont
CGS27023A + BIPBIPU
HEI
PDGFRb
/ K14
Type I
procollagen
Type V collagen
/ K14
A
B
C
D
E
F
G
H
I
50μm
50μm

Internal

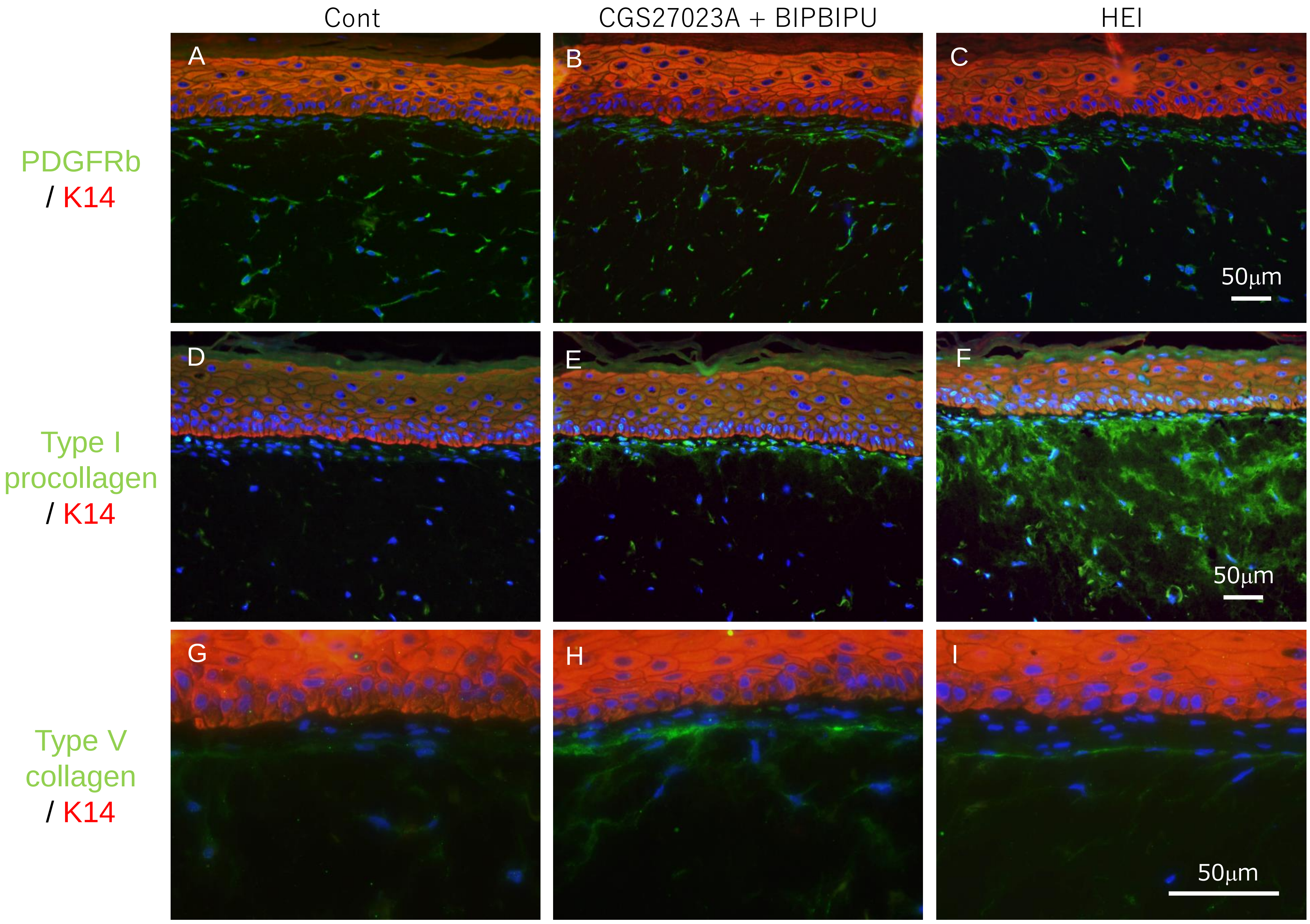

Cont
CGS27023A + BIPBIPU
HEI
PDGFRb / K14
Type I procollagen / K14
Type V collagen / K14
A
B
C
D
E
F
G
H
I
50μm
50μm
50μm

Internal

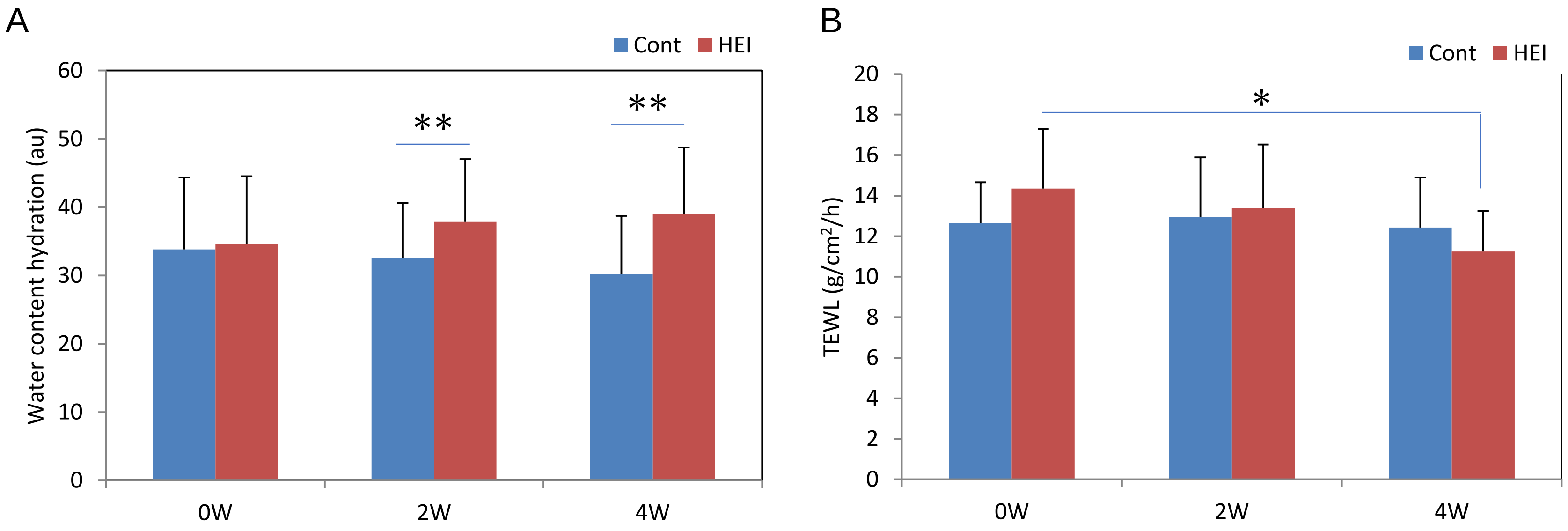
A
Cont
HEI
60
50
40
30
20
10
0
Water content hydration (au)
**
**
0W
2W
4W
B
Cont
HEI
20
18
16
14
12
10
8
6
4
2
0
TEWL (g/cm2/h)
*
0W
2W
4W

Supplementary Table S1 Source of human skin for histological experiments

Age	Sex	Origin	Anatomical site
26 years old	Female	Caucasian	Abdomen
27 years old	Female	Caucasian	Abdomen
32 years old	Female	Caucasian	Abdomen
32 years old	Female	Caucasian	Abdomen
35 years old	Female	Caucasian	Abdomen
37 years old	Female	Caucasian	Abdomen
37 years old	Female	Caucasian	Abdomen
39 years old	Female	Caucasian	Abdomen
61 years old	Female	Caucasian	Abdomen
61 years old	Female	Caucasian	Abdomen
63 years old	Female	Caucasian	Abdomen
63 years old	Female	Caucasian	Abdomen
66 years old	Female	Caucasian	Abdomen
68 years old	Female	Caucasian	Abdomen
71 years old	Female	Caucasian	Abdomen
73 years old	Female	Caucasian	Abdomen
78 years old	Female	Caucasian	Abdomen
61 years old	Female	Caucasian	Facial
61 years old	Female	Caucasian	Facial
62 years old	Female	Caucasian	Facial
66 years old	Female	Caucasian	Facial
67 years old	Female	Caucasian	Facial
71 years old	Female	Caucasian	Facial
72 years old	Female	Caucasian	Facial

Internal

Supplementary Table S2 Source of organotypic human skin

Age	Sex	Origin	Anatomical site
26 years old	Female	Caucasian	Abdomen
23 years old	Female	Caucasian	Abdomen
32 years old	Female	Caucasian	Abdomen
22 years old	Female	Caucasian	Abdomen
30 years old	Female	Caucasian	Abdomen
29 years old	Female	Caucasian	Abdomen

Internal

Supplementary Table S3 Source of cultured keratinocytes

Age	Sex	Origin	Anatomical site
12 years old	female	Caucasian	Abdomen
17 years old	female	Caucasian	Abdomen
18 years old	female	Caucasian	Abdomen
20 years old	female	Caucasian	Abdomen
22 years old	female	Caucasian	Abdomen
28 years old	female	Caucasian	Abdomen
33 years old	female	Caucasian	Abdomen
42 years old	female	Caucasian	Abdomen
50 years old	female	Caucasian	Abdomen
53 years old	female	Caucasian	Abdomen
54 years old	female	Caucasian	Abdomen
60 years old	female	Caucasian	Abdomen
62 years old	female	Caucasian	Abdomen
62 years old	female	Caucasian	Abdomen

Internal

Supplementary Table S4 Source of cultured fibroblasts

Age	Sex	Origin	Anatomical site
20 years old	female	Caucasian	Abdomen
22 years old	female	Caucasian	Abdomen
27 years old	female	Caucasian	Abdomen
28 years old	female	Caucasian	Abdomen
32 years old	female	Caucasian	Abdomen
33 years old	female	Caucasian	Abdomen
37 years old	female	Caucasian	Abdomen
50 years old	female	Caucasian	Abdomen
53 years old	female	Caucasian	Abdomen
54 years old	female	Caucasian	Abdomen
60 years old	female	Caucasian	Abdomen
62 years old	female	Caucasian	Abdomen
62 years old	female	Caucasian	Abdomen
68 years old	female	Caucasian	Abdomen

Internal
