# Supplementary material for: Regeneration of collagen fibrils at the papillary dermis by reconstructing basement membrane at the dermal–epidermal junction
Source: Sci Rep. 2022 Jan 17;12:795. doi: 10.1038/s41598-022-04856-1 (PMC8764085; doi:10.1038/s41598-022-04856-1)
Supplement: Supplementary file 2 — Supplementary Information 2. [file 41598_2022_4856_MOESM2_ESM.docx]

Regeneration of collagen fibrils at the papillary dermis by reconstructing basement membrane at the dermal-epidermal junction

Shunsuke Iriyama, Yuki Ogura, Saori Nishikawa, Junichi Hosoi and Satoshi Amano

**Supplementary Figure S1 Effect of PDGF-BB on COL5A1 expression in cultured fibroblasts**

mRNA expression levels of *COL5A1* in fibroblasts stimulated with PDGF-BB, GDNF and GM-CSF were analyzed by qPCR and normalized to GAPDH. Data are expressed as mean ± SD of six independent experiments. * P < 0.05.

**Supplementary Figure S2 Effect of PDGF-BB on accumulation of collagen fibrils at the papillary dermis in the skin equivalent model**

Immunostaining of type III pro-collagen (A-D) and type V collagen (E-H) in the skin equivalent model cultured with both MMP inhibitor CGS27023A and heparinase inhibitor BIPBIPU (B, F), or with 100 ng/mL PDGF-BB (C, G) or with the combination of 100 ng/mL PDGF-BB, MMP inhibitor CGS27023A and heparinase inhibitor BIPBIPU (D, H), as compared with the control (A, E). Bars: 100 μm.

**Supplementary Figure S3 Effect of conditioned media from cultured keratinocytes on collagen expression in cultured fibroblasts**

mRNA expression levels of *COL1A1* (A)*, COL3A1* (B) and *COL5A1* (C) in fibroblasts stimulated with conditioned media from cultured keratinocytes on plates coated with laminin-511 fragment and on non-coated plates were analyzed by qPCR and normalized to GAPDH. Data are expressed as mean ± SD of six independent experiments. * P < 0.05, ** p < 0.01.

**Supplementary Figure S4 Effect of the bifunctional inhibitor HEI on accumulation of collagen fibrils at the papillary dermis in the organotypic human skin model**

Immunostaining of PDGFR-beta (A-C), type I pro-collagen (D-F) and type V collagen (G-I) in the organotypic human skin model in the presence of both MMPs inhibitor CGS27023A and heparinase inhibitor BIPBIPU (B, E, H) or bifunctional inhibitor HEI (C, F, I), as compared with the control (A, D, G). Bars: 50 μm.

**Supplementary Figure S5 Effect of bifunctional inhibitor HEI on accumulation of collagen fibrils at the papillary dermis in the skin equivalent model**

Immunostaining of PDGFRb (A-C), type I pro-collagen (D-F) and type V collagen (G-I) in the skin equivalent model cultured with both MMP inhibitor CGS27023A and heparanase inhibitor BIPBIPU (B, E, H) or with 0.1 mg/mL HEI (C, F, I), as compared with control (A, D, G). Bars: 50 μm.

**Supplementary Figure S6 Improving effect of HEI blended lotion on water-permeable barrier function and hydration of stratum corneum of facial skin**

TEWL was measured with a vapometer before and after treatment with HEI blended lotion or with the placebo control (A). The electrical capacitance of the stratum corneum was measured with a corneometer, CM825, before and after treatment with the HEI blended lotion or with the placebo control (B). * P < 0.05, ** P < 0.01.
